# Movie Smoking and Youth Initiation: Parsing Smoking Imagery and Other Adult Content

**DOI:** 10.1371/journal.pone.0051935

**Published:** 2012-12-14

**Authors:** Matthew C. Farrelly, Kian Kamyab, James Nonnemaker, Erik Crankshaw, Jane A. Allen

**Affiliations:** 1 Public Health Policy Research Program, RTI International, Research Triangle Park, North Carolina, United States of America; 2 Community Health Promotion Research Program, RTI International, Research Triangle Park, North Carolina, United States of America; Centre for Addiction and Mental Health, Canada

## Abstract

**Objectives:**

To isolate the independent influence of exposure to smoking and other adult content in the movies on youth smoking uptake.

**Methods:**

We used discrete time survival analysis to quantify the influence of exposure to smoking and other adult content in the movies on transitioning from (1) closed to open to smoking; (2) never to ever trying smoking; and (3) never to ever hitting, slapping, or shoving someone on two or more occasions in the past 30 days. The latter is a comparative outcome, hypothesized to have no correlation with exposure to smoking in the movies.

**Results:**

Assessed separately, both exposure to smoking imagery and exposure to adult content were associated with increased likelihood of youth becoming open to smoking (OR = 1.09, 95% CI: 1.04–1.15 and OR = 1.10, 95% CI: 1.04–1.17) and having tried smoking (OR = 1.06, 95% CI: 1.00–1.12 and OR = 1.06, 95% CI: 1.00–1.13). Both measures were also separately associated with aggressive behavior (OR = 1.09, 95% CI: 1.04–1.14 and OR = 1.09, 95% CI: 1.04–1.15). A very high correlation between the two measures (0.995, p<0.000) prevented an assessment of their independent effects on smoking initiation.

**Conclusion:**

Although exposure to smoking in the movies is correlated with smoking susceptibility and initiation, the high correlation between exposure to smoking in the movies and other adult content suggests that more research is needed to disentangle their independent influence on smoking.

## Introduction

Every year, adolescents around the world are repeatedly exposed to positive images of smoking in media, most notably in movies. A survey of 137 top grossing films released in 2010 revealed that 45% of all movies; 31% of movies rated G, PG, or PG-13; and 71% of movies rated R contained tobacco imagery. [1] Since 1991, more than 650 billion portrayals of tobacco use have been transmitted to audiences in the United States through thousands of films. [2].

The pervasive use of tobacco in movies has led to an emerging literature linking exposure to multiple youth smoking behaviors. In one of the first studies to explore the issue, Dalton et al. [3] found that youth with the highest level of exposure to smoking in the movies were 2.7 times more likely than those with the lowest exposure to initiate smoking. The authors concluded that 52% of smoking initiation among youth in the cohort was attributable to exposure to smoking in the movies. Subsequent studies corroborated this association [4]–[19] or expanded on it, [20]–[23] suggesting that exposure to smoking in the movies predicts the risk of becoming an established smoker.

However, as the body of research in this area grows, our understanding of the relationship between smoking in the movies and youth behavior becomes more nuanced. For example, Primack et al. [23] and Choi et al. [24] found that exposure to movie smoking or perceptions of the amount of smoking in movies was associated with smoking behavior among those younger than age 15, but not among older youth. Several studies showed that exposure to smoking imagery in movies had a greater influence on youth who may be considered low risk for smoking based on parental smoking behavior or their level of sensation seeking. [3], [18], [20] Jackson et al. [5] and Tanski et al. [18] have demonstrated racial/ethnic differences in the association between exposure to movie smoking and subsequent youth behavior, with stronger effects among white than black youth. Dalton et al. [25] and Hanewinkel et al. [16] found that the relative risk of smoking among adolescents was significantly lower among youth whose parents restricted their movie viewing based on movie ratings.

Contributing to the complexity of research in this area is the fact that movie smoking imagery is highly correlated with other adult content, such as alcohol and other drug use, sexual situations, and violence.[16], [26]–[28] Studies report correlations ranging from.83 (for smoking and alcohol imagery) to.99 (for smoking, violence, and sexual content). [27], [28] As a result, it is difficult to disentangle the independent effect of smoking imagery on youth behavior.

Investigators conducting experimental studies have attempted to address this issue by ensuring that movie clips contain no violent or sexual content [29], [30] or by explicitly controlling exposure. [31] Shadel et al. found that exposure to smoking imagery increased the risk of smoking initiation among those with no prior risk when smoking was framed as a “social” behavior and among those already at risk when smoking was framed as a “relaxing” behavior. [29], [30] Shmueli et al. found that exposure to movie clips with smoking imagery was associated with participant smoking during a break in the study period, while exposure to the same clips with the smoking digitally removed was not. [31] A strength of these experimental studies is that they isolate the effects of smoking imagery from other adult content. However, they do not advance our understanding of the long-term effects of exposure to smoking imagery on youth tobacco use initiation and progression to established smoking.

The studies that provide the strongest data on the effect of smoking imagery on youth behavior–such as Dalton et al. [3], Sargent et al., [4] and Jackson et al. [5]–control for differences in personal characteristics such as sensation seeking, and external influences such as peer or familial smoking and parenting style, but they do not control for exposure to adult content, such as violence, profanity, or sex. We know of one study in which the authors attempted to isolate the effect of specific movie content on youth behavior. Hanewinkel et al. [27] conducted a cross-sectional survey in six European countries with 16,000 public school students (mean age of 13). The study was designed to examine the relationship between youth exposure to alcohol imagery in movies and lifetime binge drinking among adolescents. Noting the high correlation between alcohol and smoking imagery in the study movies (.83), the authors conducted a sensitivity analysis to show that while movie exposure to alcohol imagery was associated with binge drinking, exposure to smoking imagery was not. [27] Taken together, this body of work suggests that exposure to smoking imagery in movies is associated with youth smoking; what is less clear is the extent to which this relationship is causal, and the magnitude of any true causal effect.

Isolating the effect of smoking imagery in movies from other adult content would strengthen the body of research being conducted in this area, and would enable us to more precisely quantify the effect of smoking imagery on subsequent youth behavior. In this study, we measure youth’s exposure to smoking in the movies and other adult content in top grossing movies in an attempt to disentangle these separate influences on adolescent smoking initiation.

## Methods

### Data

The primary data for this study come from the New York Longitudinal Youth Tobacco Evaluation Survey (NY-LYTES). NY-LYTES is designed to capture youth smoking transitions, from never smoking to experimentation and regular smoking, and the factors that influence these transitions, with a special focus on exposure to smoking in the movies. We conducted a random-digit-dial telephone survey of 1,511 New York State youth, aged 13 to 16 at baseline, in the spring and summer of 2005. Subsequent annual follow-up surveys included 1,060 youth in 2006, 809 youth in 2007, and 632 youth in 2008. NY-LYTES and the process of obtaining consent/assent for participation were both approved by the RTI International Institutional Review Board (IRB) and the New York State Department of Health IRB. Informed consent was provided orally by parents of survey respondents; respondents orally provided assent to participate. Oral rather than written consent/assent was obtained because the survey was conducted by telephone only. Oral consent/assent was documented by the interviewer as approved by the IRB. Key constructs are described below.

### Outcome Measures

Outcomes are event indicators that indicate transitions from (1) closed to open to smoking; (2) never to ever trying smoking; and (3) never to ever hitting, slapping, or shoving someone on two or more occasions in the past 30 days. An indicator of *ever smoking* is based on an affirmative response to the question, “Have you ever tried cigarette smoking, even 1 or 2 puffs?” *Openness to smoking* is assessed only among those who report having never smoked a cigarette, even one or two puffs, and measured using three items on a 5-point Likert scale (definitely yes, probably yes, probably no, definitely no, and no opinion): (1) Do you think you will try a cigarette soon?; (2) Do you think you will smoke a cigarette at any time during the next year?; and (3) If one of your best friends offered you a cigarette, would you smoke it? Youth are considered closed to smoking (0) if they respond “Definitely not” to all three items and open to smoking (1) otherwise. [32] Our final outcome is based on the question, “During the past 30 days how many times did you hit, slap, or shove someone?” with responses of never, once, twice, or three or more times. Based on this question, we created an indicator for hitting, slapping, or shoving someone on two or more occasions (1) or less than two (0) occasions.

### Potential Influences

In our analyses, we account for many of the same potential influences used in related literature on smoking in the movies, [25], [33], [34] including sensation seeking, receptivity to tobacco marketing, and parental monitoring of and rules about watching R-rated movies.

#### Exposure to smoking in the movies

To estimate youth’s exposure to smoking in the movies, we combined data on top grossing films from www.boxofficemojo.com with rating information on the amount of smoking in movies from SceneSmoking.com, and youth self-reports on seeing individual movies. We first, we identified the top domestic grossing movies for the year preceding the annual wave of the survey. For the baseline wave of the survey, we selected 10 of the top grossing films with no or some tobacco use and 20 of the top grossing films with high levels of smoking. For subsequent waves of the survey, we selected 15 movies: 2 with little or no smoking and 13 with a lot of smoking. Of the 71 unique movies across all years, 68% (48) are PG-13, 18% (13) are R, 11% (8) are PG, and 3% (2) are G. Movies with no smoking received a score of 1 (N = 10); minimal amount of smoking, 2 (N = 4); moderate amount of smoking, 3 (N = 15); and a significant amount of smoking, 4 (N = 42). Based on these ratings, we created the Smoking in the Movies Exposure (SME) index. This index was calculated by multiplying a movie’s smoking rating by a number that indicates whether an individual has never seen the movie (0), has seen the movie only once (1), or has seen it more than once (2) and then summing this score across all movies in each wave. Individuals’ exposure for each given wave was then summed across all preceding waves to create a continuous, cumulative measure of exposure to smoking in the movies. This cumulative measure was scaled down by a factor of 20 to facilitate the interpretation of odds ratios.

#### Exposure to adult content in the movies

To estimate youth’s exposure to adult content, we used ratings assigned by Kids-in-Mind.com, a Web site operated by the for-profit company Critics, Inc. The Kids-in-Mind rating system provides more detailed information on adult movie content than the system developed by the Motion Picture Association of America. While the Motion Picture Association assigns movies a single rating such as G, PG, or R, Kids-in-Mind assigns a 0 to 10 rating for three categories of adult content: sex/nudity, violence/gore, and profanity. Based on these data, we constructed the Adult Content Exposure (ACE) index in a similar manner to the SME index. We multiplied the Kids-in-Mind scale (0 to 30) by the frequency indicator (0, 1, 2) and then summed across all movies in each wave. We also created a cumulative sum at each wave to incorporate exposure in previous waves. The cumulative measure was then scaled down by a factor of 100.

#### Sensation seeking

We measured sensation seeking, which has been shown to be associated with youth smoking, with the Brief Sensation Seeking Scale items: (1) I would like to explore strange places; (2) I like to do frightening things; (3) I like new and exciting experiences, even if I have to break the rules; and (4) I prefer friends who are exciting and unpredictable. [35] The items use a 5-point Likert scale (1 = strongly agree, 5 = strongly disagree). These responses were then reverse coded such that higher scores indicated stronger agreement. The four items were then summed, and a dichotomous indicator was created to indicate whether a respondent was below the median of this scale (0) or above (1). Youth missing data for any of the four items were considered to have missing data for this measure.

#### Tobacco marketing receptivity

To measure youth’s receptivity to tobacco marketing, we constructed a receptivity score with 0 indicating that an individual was unable to name a brand of cigarettes, 1 indicating that they were able to name a brand of cigarettes, 2 indicating that they had a favorite cigarette advertisement (as well as being able to name a cigarette brand), and 3 indicating that they owned and were willing to use merchandise bearing a cigarette company’s name or logo (in addition to being able to name a cigarette brand and having a favorite ad).

#### Parental rules for and monitoring of R-rated movie and television watching

We accounted for restrictions imposed by parents on respondents’ viewing of R-rated movies with two measures based on two questions with 4-point Likert scale response options (1 = never, 4 = all the time): (1) How often do your parents let you watch movies or videos that are rated R?; and (2) When you watch an R-rated movie, how often do you watch it with one or both of your parents? The first dichotomous measure equals 1 if the parent allows viewing R-rated movies “most of the time” or “all of the time” and 0 otherwise. The second measure is 1 if the parent “never” or “sometimes” co-views and is 0 otherwise.

Parental oversight and involvement in respondents’ movie viewing was assessed in a manner detailed by Dalton et al. [25] Four items on a 4-point Likert scale (1 = never, 4 = all the time) were used: (1) How often do your parents want to know what a movie is rated before you can see it?; (2) How often do you have to check with your parents before watching a movie?; (3) How often do your parents go into the video store with you when you rent a movie?; and (4) When you go to a friend’s house, how often does your parent check to see what movie you might be watching? The final two items (3, 4) allowed for youth to respond that they “do not go to the movie store” and “do not go to friends’ houses,” respectively. Responses were dichotomized so that responses other than “All the time” equaled 0.

Parental limits on television viewing were measured by responses to the question, “Do your parents limit the amount of time you spend watching TV or movies?” Responses were dichotomous, and respondents were coded with a 1 for “yes” responses and a 0 for “no” responses.

#### Other variables

Other measures used for analysis include age, race/ethnicity, gender, residence in New York City, attendance of a public school, a dichotomized indicator for above average academic achievement, church attendance, the presence of an adult at home after school, employment, income (scaled down by a factor of 100), having a friend who smokes, having a friend who smokes marijuana, presence of a smoking ban in respondent’s household, exposure to secondhand smoke, and exposure to tobacco use prevention lessons in school.

### Analysis

We estimated the relationship between exposure to smoking in the movies and youth transitions using discrete-time survival analysis. To use discrete-time survival analysis requires creating a person-year data set in which each individual contributes to the estimation as long as he or she is at risk of the event occurring. That is, at each age, we start with all sample members who have not experienced the event but are at risk of the event occurring (e.g., never smoked in the model of the transition to ever smoking) and then estimate the risk of the event occurring (transition to smoking) as the sample of youth ages.

We used a logistic regression model to estimate the probability of event occurrence given that the event has not yet occurred. [36], [37] In these models, once the event in question occurs, the sample member is dropped from subsequent time periods because he or she is no longer at risk. This process allowed the calculation of the odds that an individual will initiate smoking for each age represented in the sample, given that they had not begun smoking previously. Although there is attrition from the cohort over time, the sample size used in these analyses depends on whether the event occurred during any of the surveys in which the youth participated.

The key covariates of interest are the SME and ACE indexes. For each outcome, we estimated separate models using either the SME or ACE index. For all regressions, we present two model specifications: (1) a crude, bivariate model and (2) an adjusted model including the covariates described above. In addition to these models, we examined the degree of correlation between the SME and ACE indexes.

As a test of whether exposure to smoking in the movies is a proxy for youth’s risk more broadly, rather than for smoking initiation specifically, we also examined whether exposure to smoking in the movies is correlated with youth exhibiting aggressive behavior.

## Results

Table 1 presents descriptive statistics for the baseline sample of youth. At baseline, observed SME scores ranged from 0 to 174, with a mean score of 70.6 out of a total possible score of 180. ACE scores ranged from 0 to 724, with a mean score of 304.1 out of a total possible score of 760. Table 2 illustrates the number of person-years of data available for each outcome, the number who experience the event of interest (i.e., “fail”), and the number of youth who are right censored (i.e., lost to follow-up before experiencing the event/outcome of interest).

**Table 1:** Baseline Demographics and Summary Statistics from the New York Longitudinal Youth Tobacco Evaluation Survey (NY-LYTES), 2005–2008.

Measure	n = 1511
Age (Years)	
13	21.1%
14	26.3%
15	28.5%
16	24.1%
Race/Ethnicity	
Non-Hispanic White	85.4%
Non-Hispanic African American	4.6%
Hispanic	3.3%
Other Race	6.3%
Gender	
Male	53.4%
Female	46.6%
New York City resident	11.3%
Sensation seeker (proportion of cohort above median)	48.6%
Attend public school	84.5%
Above average student	55.4%
Attend church frequently	53.6%
Adult at home after school	77.8%
Employed	22.4%
Monthly income/allowance (scaled)	$31.39 ($0.31)
One or more friends smoke cigarettes	31.6%
One or more friends smoke marijuana	28.0%
Smoking ban in household	76.7%
Number of days exposed to secondhand smoke in the past 7 days	1.42
Smoker in household	26.3%
Tobacco use prevention education in school	41.6%
Parents limit television viewing	37.4%
Parents permit R-rated movie viewing most of the time/always	45.9%
Parents co-view R-rated movies never/sometimes	74.4%
Parents want to know movie rating before viewing	31.6%
Must check with parents before watching a movie	16.6%
Parents go to the video store when renting movie	29.3%
Parents check on which movies might be seen at friend’s home	4.6%
Tobacco marketing receptivity	
None	33.2%
Low	23.1%
Medium	21.1%
High	22.6%
Ever smoked	18.1%
Open to smoking	30.3%
Exhibited aggressive behavior	26.1%
Mean SME index (not scaled)	3.5 (70.6)
Mean ACE index (not scaled)	3.1 (304.5)

Note: ACE = Adult Content Exposure; SME = Smoking in the Movies Exposure.

**Table 2:** Life Tables for the New York Longitudinal Youth Tobacco Evaluation Survey (NY-LYTES), 2005–2008.

Never to Ever Smoking					Closed to Open to Smoking				No Aggressive Behavior to 2 or More Instances			
Age	Beginning Sample	Fail	% Fail	Right-Censored	Beginning Sample	Fail	% Fail	Right-Censored	Beginning Sample	Fail	% Fail	Right-Censored
13	1,394	0	0%	83	1,286	0	0%	64	1,404	1	0%	67
14	1,311	21	2%	122	1,222	56	5%	94	1,336	70	6%	102
15	1,168	58	6%	156	1,072	81	8%	118	1,164	111	11%	149
16	954	113	16%	246	873	81	12%	204	904	119	18%	241
17	595	115	31%	219	588	72	20%	236	544	99	29%	199
18	261	37	33%	148	280	18	17%	171	246	17	16%	141
19	76	9	53%	59	91	4	36%	80	88	5	45%	77
20	8	3	100%	5	7	0	n/a	7	6	1	100%	5

“Fail” is the number of youth who experience the event of interest; “Right-Censored” is the number lost to follow-up before experiencing the event/outcome of interest.

Exposure to smoking in the movies is significantly associated with increased odds of never smokers becoming open to smoking and initiating smoking (Table 3). We find that as the SME index increases, youth are more likely to be open to smoking (adjusted OR = 1.09, 95% CI: 1.04–1.15) and to have tried smoking (adjusted OR = 1.06, 95% CI: 1.00–1.12). Because the SME index is a continuous variable, another method for understanding the magnitude of the effect is to compare observed smoking initiation with predicted smoking initiation assuming the SME index is zero. This comparison indicates that smoking initiation would have been 21% lower if this cohort was never exposed to smoking in the movies for the fully adjusted model and 66% lower for the simple bivariate model. In addition, increases in the SME index are associated with increases in the likelihood of engaging in aggressive behavior (adjusted OR = 1.09, 95% CI: 1.04–1.14).

**Table 3:** Influence of Exposure to Smoking in the Movies and Adult Content on Initiation to Smoking, Becoming Open to Smoking, and Exhibiting Aggressive Behavior.

	Cumulative Exposure to Smoking in Movies		Cumulative Exposure to Adult Content in Movies	
Transition	Crude	Adjusted	Crude	Adjusted
Closed to open to smoking Person-year observations	1.15*** (1.11–1.20) 2,589	1.09*** (1.04–1.15) 2,474	1.17*** (1.12–1.22) 2,589	1.10*** (1.04–1.17) 2,474
Never to ever smoked (95% CI) Person-year observations	1.23*** (1.19–1.27) 3,456	1.06** (1.00–1.12) 3,307	1.27*** (1.22–1.32) 3,456	1.06* (1.00–1.13) 3,307
No aggressive behavior to 2 or moreinstances Person-year observations	1.15*** (1.12–1.19) 3,407	1.09*** (1.04–1.14) 3,261	1.17*** (1.13–1.21) 3,407	1.09*** (1.04–1.15) 3,261

*** p<0.01.

** p<0.05.

* p<0.1.

Next, we replaced the SME index with the ACE index and find similar results (see Table 3). As this index increases, youth are more likely to be open to smoking (adjusted OR = 1.10, 95% CI: 1.04–1.17) and to have tried smoking (adjusted OR = 1.06, 95% CI: 1.00–1.13). Additionally, increases in the ACE index are associated with increases in the likelihood of engaging in aggressive behavior (adjusted OR = 1.09, 95% CI: 1.04–1.15).

**Figure 1:**
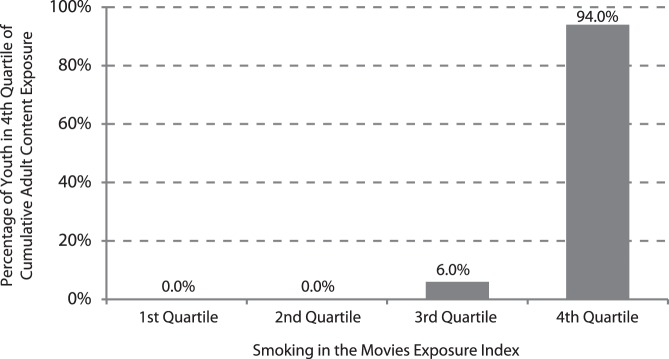
Percentage of the New York Longitudinal Youth Tobacco Evaluation Survey (NY-LYTES) 2005–2008 Sample in the Highest Quartile of Exposure to Adult Content in Movies for each Quartile of Exposure to Smoking in Movies.

Given the similarity of the results for the SME and ACE indexes, we examined the correlation between the two indexes and found that it is 0.995 (p<0.001). The similarity between the two measures is illustrated in Figure 1. For this figure, each sample member is categorized into quartiles of exposure to smoking in the movies and adult content. We then display the percentage of the sample that is in the highest quartile of adult content for each of the quartiles of exposure to smoking in the movies. This shows that nearly all the youth who are in the highest quartile for exposure to adult content are also in the highest quartile for exposure to smoking in the movies. Additionally, we estimated the correlation between modified versions of the indexes at baseline by recalculating SME and ACE indexes using a dichotomous, as opposed to a three-level, frequency of exposure indicator. At baseline, the correlation between the recalculated indexes is 0.992 (p<0.001).

## Discussion

This study was designed to build on earlier work examining the relationship between exposure to smoking imagery in movies and youth smoking. Noting the correlation between smoking imagery and adult content in movies, the intent of this analysis was to illustrate that exposure to smoking imagery was correlated with smoking initiation *above and beyond* any potential influence of other adult content. We sought to disentangle the influence of smoking imagery from other adult content–such as nudity, profanity, and violence–by creating separate exposure measures for each. Based on a sample of 71 top grossing films over a 4-year period, we found that the correlation between smoking imagery and other adult content was so high that it was impossible to disentangle their separate influence. In fact, the two measures are almost perfectly correlated.

The link between youth exposure to smoking in movies and subsequent smoking behavior has been observed in numerous studies, using different designs, and in multiple countries. Based on theories of social learning, [38] it is plausible that exposure to smoking imagery in movies influences youth smoking behavior. However, other theories, such as Problem Behavior Theory, [39], [40] would also lead one to postulate a plausible relationship between exposure to other adult content and smoking. Although studies have attempted to control for variables that might be common to both exposure to smoking in movies and smoking behavior (e.g., sensation seeking), biases in the measurement of these variables (due to youth self-reports for example) could still result in a biased estimate of the relationship between exposure to smoking in the movies and smoking behavior.

This study raises at least three challenges to investigators working in this area. First, we should attempt to disentangle the effects of exposure to smoking imagery from other adult content to better quantify the independent influence of smoking in the movies. Experimental studies such as the one conducted by Shmueli et al. [31] have begun to address this issue. Investigators with more extensive data sets and a greater selection of movies should also be able to improve upon existing research using survey data to disentangle the effects of exposure to smoking in the movies from other adult content.

Second, studies that do not randomly assign adolescents to movie exposure should make every effort to control for youth self-selection of adult content. It is possible that youth who elect to watch movies with smoking and other adult content are also more likely to smoke; in other words, the apparent relationship is due, at least in part, to some unmeasured variable or variables. To date, studies have attempted to control for factors that may be associated with increased exposure to adult content, such as sensation seeking, parenting style, and peer or parent smoking. However, there may be room for improvement in this area; at least one study shows that common measures of sensation seeking do not perform equally well across race-ethnicity, [41] and youth self-reports of parenting variables may be prone to bias. [42] Third, we should begin to develop a better sense of factors that may mediate or moderate the relationship between exposure to smoking imagery and youth behavior. For example, new research suggests that youth who are more highly transported into narratives show greater attitude, belief, and behavior change as a result of those narratives, a finding that may have important implications for our work. [43] Investigators in tobacco control and public health should work collaboratively with colleagues in communication and media research to identify additional potential factors of importance to this body of research.

This study has several limitations. First, the study is based on a smaller set of movies than the seminal work by Sargent, Dalton, and colleagues. Second, the current study examines youth who were older at baseline (aged 13 to 16) than in previous studies, potentially missing earlier initiation. Third, this study relies on data that were not collected or analyzed by the investigators, and for which we have no information related to reliability or validity: boxofficemojo.com (to define top grossing movies), SceneSmoking.com (to develop the SME index), and Kids-in-Mind.com (to develop the ACE index). Fourth, the telephone surveys used in this study likely underestimate the true level of smoking compared to other modes of data collection (e.g., school, household). [44] Despite these limitations, our results are qualitatively similar to many other similar studies, although we draw different conclusions.

This study raises questions about the extent to which existing studies have established a causal link between smoking in movies and youth behavior. Our findings suggest that additional studies are needed to disentangle exposure to smoking imagery and other adult content so as to quantify the magnitude of a true causal relationship between exposure to smoking in the movies and youth smoking.
